# Warburg effect in B-cell lymphoma: A case report and proposed management plan

**DOI:** 10.2478/jccm-2025-0045

**Published:** 2026-01-30

**Authors:** Stefan Gligor, Salim Abdelhamid, Veronika Ballova, Andrea Kopp Lugli

**Affiliations:** Baden Cantonal Hospital, Baden, Switzerland

**Keywords:** intensive care, oncology, hematology, lymphoma, lactic acidosis, metabolic acidosis

## Abstract

**Introduction:**

The Warburg effect is a rare but often fatal condition in patients with malignancies. This phenomenon, known as type B lactic acidosis, is defined by lactatemia without tissue hypoxia or hypoperfusion, in contrast to type A lactic acidosis, which usually results from either or both.

**Case presentation:**

A male patient in his seventies with a newly diagnosed diffuse large B-cell lymphoma is admitted to the intensive care unit due to severe metabolic derangements with hypoglycemia and lactatemia. Extensive investigations ruled out alternative etiologies, strongly suggesting the Warburg effects as the underlying mechanism. Despite hemodynamic instability, chemotherapy was initiated and resulted in initial clinical improvement.

**Conclusion:**

We propose a stepwise approach to improve the management of patients with suspected type B lactic acidosis.

## Introduction

The Warburg effect proposes that malignant cells exhibit a unique metabolic preference for glycolysis even in the presence of ample oxygen [1,2,3]. Originally hypothesized to result from irreparable damage to respiratory processes, the modern interpretation of the Warburg effect highlights its significance in cancer, emphasizing the prioritization of dysregulated glycolysis rather than compromised respiration [1, 4]. This phenomenon, also referred to as type B lactic acidosis (LA), is mostly described in patients with aggressive tumors and characterized by LA in the absence of tissue hypoxia or hypoperfusion [5]. While the Warburg effect has been extensively studied in the context of experimental research, its implications in the clinical setting have received increasing attention, especially in the field of intensive care. It creates a metabolic picture that closely mimics other diseases found in the ICU, including tumor lysis syndrome and sepsis-associated lactic acidosis. Distinguishing this malignancy-driven process from other causes of metabolic derangements and forms of LA encountered in the critically ill setting is, therefore, a crucial, yet challenging aspect of modern critical care [6, 7]. The complexity of etiologies induces diagnostic and therapeutic challenges that we aim to address by exploring a case of a critically ill patient with a recently diagnosed lymphoma.

## Case presentation

A 73-year-old male patient was urgently referred by his primary care provider to our hospital with a three-month history of progressive fatigue, fevers, and unintentional weight loss exceeding 10%. His medical history revealed biopsy-confirmed hepatic siderosis without evidence of genetic hemochromatosis, alongside a prior Hepatitis B infection.

Chest, neck, and abdominal computed tomography (CT) showed cervical and inguinal lymphadenopathy, multiple pulmonary lesions, and significant hepatosplenomegaly. Laboratory findings included thrombocytopenia as well as elevated lactate dehydrogenase (LDH), aspartate and alanine transaminases (AST, ALT), alkaline phosphatase, creatinine and C-reactive protein (CRP), as detailed in **Table 1**, which also presents the laboratory values at peak lactatemia — a constellation consistent with developing multi-organ failure.

**Table 1.** Laboratory values at hospital admission and at peak lactate level

**Laboratory parameter**	**Value at hospital admission**	**Value at peak lactate**	**Normal reference range**
Hemoglobin	14.6 g/dL	11.0 g/dL	14.1 – 17.5 g/dL
Thrombocytes	89’000/μL	65’000/μL	150’000 – 450’000/μL
White blood cells	6’720/μL	10’200/μL	3’700 – 11’200/μL
CRP	80 mg/L	45.6 mg/L	< 5 mg/L
Creatinine	1.31 mg/dL	1.71 mg/dL	0.7 – 1.2 mg/dL
Potassium	4.4 mmol/L	4.3 mmol/L	3.5 – 4.9 mmol/L
Phosphate	4.1 mg/dL	3.8 mg/dL	2.5 – 4.5 mg/dL
Uric acid	7.9 mg/dL	3.0 mg/dL	3.4 – 7.0 mg/dL
ALT	61 U/L	116 U/L	10 – 50 U/L
AST	86 U/L	587 U/L	10 – 50 U/L
Alkaline phosphatase	175 U/L	415 U/L	40 – 130 U/L
Bilirubin	0.9 mg/dL	3.0 mg/dL	< 1.2 mg/dL
Lactate dehydrogenase	653 U/L	3343 U/L	135 – 225 U/L
Lactate	NA	17 mmol/L	0.5 – 1.6 mmol/L

All numbers rounded to the nearest integer. Bold values are not within the normal reference range. NA = not available; lactate was not tested for in the routine laboratory assessment at hospital admission

Histological examination of bone marrow and lymph node samples, obtained via biopsy and guided excision, found widespread abnormal cell activity. A positron emission tomography (PET) scan confirmed metabolically active lesions in the chest, abdomen, and spleen. Initial findings suggested diffuse large B-cell lymphoma, later confirmed by further analysis as the non-germinal-center subtype (proliferation fraction 95%, EBV negative, BCL6 rearrangement, Ann Arbor stage 4B, international prognostic index 4 [high risk] [8]).

High-dose prednisolone (50mg/day; Streuli Pharma, Switzerland) was started to prepare the patient for chemotherapy, while tenofovir (25mg/day; Gilead Sciences, Switzerland) guarded against hepatitis B reactivation. Due to elevated uric acid indicating high risk for tumor-lysis syndrome, allopurinol (300mg/day; Helvepharm, Switzerland) was initiated for prophylaxis. Ten days into hospitalization and five days on high-dose steroids, the patient rapidly declined with increasing somnolence and muscle weakness. Tests revealed high-grade acute kidney failure (creatinine 2.6 mg/dL), severe metabolic acidosis (pH 7.263, lactate 7.1 mmol/L), hypoglycemia (glucose 3.1 mmol/L) and hypoxic partial respiratory failure (pO_2_ 77.3 mmHg). A CT scan showed bilateral pleural effusions, potential signs of infection, and significant tumor growth in the interim.

Progressive multiorgan failure necessitated the transfer to the intensive care unit (ICU). Hemodynamic stabilization was achieved with norepinephrine, while further respiratory deterioration was addressed via invasive mechanical ventilation (with maximal support required during the initial phase: DuoPAP mode, FiO_2_ 45%, respiratory rate 18/min, PEEP 8 mbar and P(peak) 28 mbar with target PaO_2_ of > 60 mmHg and target etCO_2_ between 33.7 and 45 mmHg, P(A-a) gradient 168 mmHg). Continuous veno-venous hemofiltration (CVVH) was initiated for refractory acidosis, accompanied by empirical broad-spectrum antibiotics (piperacillin-tazobactam; Sandoz, Switzerland) targeting suspected septic shock. To expedite glucocorticoid delivery and ensure adequate absorption, intravenous methylprednisolone (250mg/day; Pfizer, USA) replaced the oral administration. Metabolic correction involved 5% glucose and sodium bicarbonate infusions, bolstered by intermittent 20% glucose boli. However, treatment-resistant lactatemia and relapsing hypoglycemia necessitated further intervention. Additional vasopressin administration (0.01 units/min at a rate of norepinephrine 39 μg/min), plasma expansion with 20% albumin (CSL Behring, Switzerland), antibiotic escalation to meropenem (Fresenius Kabi, Switzerland) and vancomycin (Labatec Pharma, Switzerland) and sustained pH normalization via CVVH further stabilized hemodynamics but failed to resolve metabolic derangements. Medical nutrition therapy was started after stabilization on ICU day three according to the ESPEN guideline for critically ill patients [9] with Fresubin Intensive^®^ (1.2 kcal/ml, 10 g protein/100ml; Fresenius Kabi, Switzerland) at a rate of 10 ml/h, which was steadily increased to reach the calculated energy (20 kcal/kg) and protein target (1.3g/kg) at ICU day 6. The calculated energy target was measured and adapted by indirect calorimetry at ICU day 5 resulting in resting energy expenditure of 1348 kcal.

After exclusion of differential diagnoses for lactatemia (see **Table 2**), the malignancy itself was identified as the most plausible explanation for the observed metabolic derangements, potentially through pathways of the Warburg effect. Notably, propofol was administered during the ICU stay, however, only after the lactate had peaked, making a propofol-induced lactatemia highly unlikely. Other medications commonly implicated in causing lactic acidosis, including salicylates and metformin, were not part of the patient’s treatment.

**Table 2.** Diagnostic approach to metabolic derangements

**Possible cause of lactatemia**	**Reason for exclusion**
Hepatic failure	Adequate hepatic function in clinical and laboratory testing (bilirubin, INR, albumin, ascites, ammonium)
Hypoperfusion	Adequate cardiac output in invasive arterial monitoring
Sepsis	Persistence of disturbances after initiation of broad-spectrum antibiotics without evidence of resistant organisms in cultures, clinically apparent infection or signs of infection in diagnostic imaging
Respiratory failure	Persistence after adequate oxygenation and respiratory support
Thiamine-deficiency	Persistence after thiamine substitution (300mg/day)
Propofol-induced lactic acidosis	Persistence after switch to benzodiazepine-based sedation
D-lactic acidosis	Normal d-lactate in laboratory testing
Medication or toxins	No exposure to possible causative agents

**Fig. 1.**
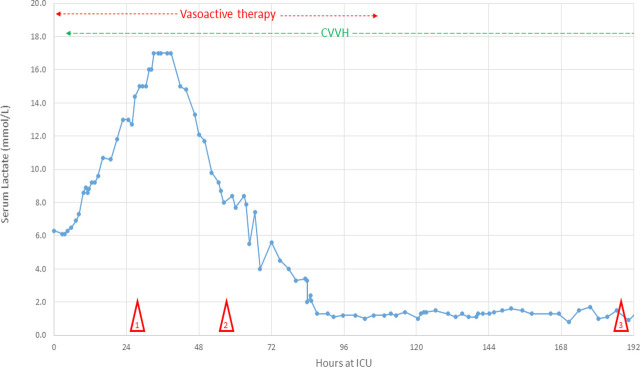
**Course of serum lactate during ICU stay.** Course of serum lactate, peaking at 17 mmol/L on the second day in the ICU. Vasoactive therapy was discontinued on ICU day five, CVVH on ICU day nine. Mark 1: initiation of chemotherapy; mark 2: onset of disseminated intravascular coagulopathy; mark 3: extubation

Despite maximal supportive care, the metabolic imbalance persisted with worsening lactatemia (see **Figure 1**). Given the critical condition, the interdisciplinary decision for early initiation of chemotherapy was made. Treatment commenced on ICU day two, administering rituximab (Roche, Switzerland) and cyclophosphamide (Baxter, Switzerland) on day one and two. Vincristine and doxorubicin were stopped to minimize further hepatotoxicity.

On day two of chemotherapy a favorable response was observed, with lactate and glycemic levels normalizing by day three. However, the clinical course remained challenging: On day three the patient endured disseminated intravascular coagulopathy (DIC) manifested as recurrent catheter-associated bleeding and thrombosis. Management involved multiple platelet transfusions, administration of tranexamic acid (Viatris, USA), and factor XIII guided (CSL Behring, Switzerland) by serum fibrinogen, platelet count and the clinical course. Following resolution of the inflammatory state and cessation of bleeding, slowly titrated therapeutic anticoagulation with heparin was initiated to prevent thrombi.

Seven days post-induction, neutropenia developed, prompting a seven-day course of granulocyte colony-stimulating factor (G-CSF; Sandoz, Switzerland). Concomitantly, the patient exhibited remarkable clinical improvement, resulting in ventilator and vasopressor weaning within ten days of ICU admission.

Renal function also began to recover and CVVH was discontinued two weeks after ICU admission. Following stabilization, the patient was transferred to the medical ward. Prior to discharge for rehabilitation, a second cycle of R-CHOP (rituximab, cyclophosphamide, doxorubicin, vincristine and prednisone) chemotherapy was administered.

### Follow-up

A brief post-discharge period culminated in rehospitalization for Clostridioides difficile colitis, effectively treated with oral vancomycin. During this admission, the third cycle of R-CHOP was administered, followed by discharge to rehabilitation.

Still, subsequent PET-CT imaging revealed disease progression despite three completed chemotherapy cycles. Chimeric antigen receptor T-cell therapy was planned as salvage therapy.

In the interim, however, the patient’s clinical status deteriorated following discharge from rehabilitation. This necessitated a final hospitalization due to progressive fatigue, weakness, and abdominal pain. Clinically, the patient exhibited rapid malignant progression, characterized by the development of malignant ascites and recurrence of the previously documented metabolic derangements (lactatemia and hypoglycemia). These derangements were again suspected to be a manifestation of the Warburg effect.

In light of the patient’s progressive disease, a shared decision to cease further treatment was made. The patient passed away shortly thereafter.

## Discussion

Warburg effect induced type B lactatemia represents a rare but important differential diagnosis of metabolic derangement in critically ill patients. While most cases of LA can be attributed to type A lactatemia in septic, hypovolemic shock or respiratory failure, it remains an important diagnosis to consider in patients, whose metabolic disturbances persist after adequate hemodynamic and respiratory stabilization [10]. The Warburg effect is mostly described in patients with aggressive tumors and characterized by increased levels of lactate in the absence of tissue hypoxia or hypoperfusion. Originally hypothesized to result from irreparable damage to respiratory processes, the current interpretation of the Warburg effect highlights its significance in cancer, emphasizing the prioritization of dysregulated glycolysis rather than compromised respiration. While normal cells release energy through the mitochondrial citric acid cycle and oxidative phosphorylation, many cancer cells rely heavily on glycolysis, even in oxygen-rich environments, leading to lactic acid fermentation. This anaerobic glycolysis is significantly less efficient for adenosine triphosphate production than oxidative phosphorylation but results in the generation of metabolites that can advantageously support cancer cell proliferation [1, 4, 5, 11]. This mechanism differentiates the Warburg effect induced LA from other type B variants, where the increase of serum lactate is caused by inadequate clearance of physiologically produced lactate. Hence, type B LA warrants consideration in patients with established or suspected hematologic malignancies and, less frequently, in solid tumors [12]. Consequently, its presence with hypoglycemia should raise a strong suspicion for the Warburg effect [13].

The differentiation between malignancy associated LA and other causes should be pursued aggressively to initiate adequate treatment in a timely fashion. Distinguishing between the two types of LA can be achieved by assessing persistence of lactatemia after stabilization of cardiovascular and respiratory functions using vasopressors, volume expansion and/or respiratory support if necessary. Sepsis as a possible contributor to acidosis should be ruled out if possible by microbiologic cultures and diagnostic imaging. A pre-emptive treatment with broad-spectrum antibiotics and antifungal medication should be initiated in case sepsis cannot be excluded completely. However, hematologic malignancies inherently render patients immunocompromised, thereby increasing their susceptibility to develop fulminant sepsis, particularly from fungal pathogens [14]. Furthermore, the unreliable utility of inflammation markers like CRP and WBC in this population complicates the differential diagnosis between sepsis- and malignancy-induced acidosis. This diagnostic challenge is compounded by emerging data that the Warburg effect is not exclusive to cancer but is also integral to the metabolic reprogramming seen in sepsis itself [6]. This shared pathophysiology provides a basis for the clinical mimicry between these two life-threatening conditions. Also, acute hepatic failure can present similarly with type B lactic acidosis and spontaneous hypoglycemia along with several electrolyte imbalances [15]. Evaluation of liver function parameters is crucial for achieving an adequate distinction whether the observed metabolic abnormalities can be attributed to significant hepatic dysfunction [16].

Treatment of this rare cause of LA is a significant challenge, further compounded by the exceedingly high mortality rate of over 90% [17, 18]. While no definitive guidelines have been established, a review of published case reports reveals multiple commonalities in terms of therapeutic approach. Initial management typically involves hemodynamic stabilization with volume resuscitation and vasopressor support to address vasoplegia and the resulting reduction in cardiac output due to acidosis [12, 19, 20]. Early implementation of respiratory support is also frequently indicated due to metabolic acidosis-induced hyperventilation and consequent respiratory fatigue. Metabolic correction is often attempted by combining sodium bicarbonate infusion and renal replacement therapy [21, 22]. However, data regarding the survival benefits of these measures remain limited. Notably, some case reports suggest that large doses of sodium bicarbonate infusion might even stimulate lactate production indirectly through fluid overload and intracellular acidification by accumulation of CO_2_ [22]. Therefore, these interventions may primarily serve as a bridge until definitive and only causative therapy for malignancy associated LA in form of cytoreductive agents can be initiated. This principle is strongly supported by a larger cohort study, which found that despite often not requiring advanced organ support, prompt initiation of chemotherapy is essential in this vulnerable patient group [7]. However, patient tolerance to chemotherapy may be compromised by poor overall condition, electrolyte imbalances, the underlying acidosis itself, renal dysfunction or hepatic impairment. Early and aggressive interventions to restore homeostasis are crucial to facilitate the initiation of chemotherapy. Early involvement and interdisciplinary cooperation between the intensive care team and oncology are also essential to determine optimal chemotherapy regimen considering the underlying pathology and the patient’s tolerability for chemotherapy. Thus, even after initiation of adequate chemotherapy patients suffering from malignancy-associated LA remain at high risk.

Hematologic malignancies pose a unique challenge due to the inherent myelotoxicity of many chemotherapeutic agents. This may lead to a cascade of complications: Thrombocytopenia increases the risk for bleeding events, while agranulocytosis significantly elevates the susceptibility to infections. Additionally, anemia can worsen peripheral oxygen delivery, further compromising organ function. Initiation of chemotherapy can also trigger tumor lysis syndrome (TLS). This rapid breakdown of cancer cells releases intracellular ions into the bloodstream, potentially causing severe electrolyte imbalances, acute kidney injury and even worsening LA. The risk of TLS is particularly concerning for malignancies that overlap with those predisposed to malignancy-associated lactic acidosis [23]. Consequently, the utility of LDH in this differential diagnosis is limited, as it lacks specificity to reliably distinguish between the Warburg effect and TLS. Given the risk of TLS, vigilance, early detection and prompt management are crucial components of treatment for these patients.

**Fig. 2.**
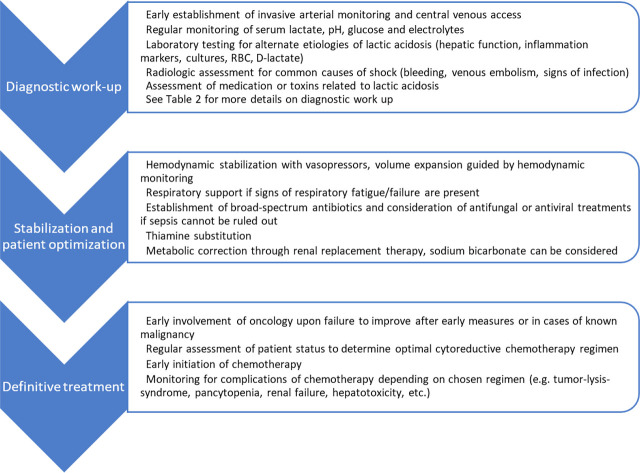
Proposed management algorithm

The intricate clinical presentation and high mortality of Warburg effect-induced LA underscore the need for a structured diagnostic and therapeutic approach. To facilitate prompt and appropriate management in this complex patient group, we propose a stepwise plan that integrates diagnostic work-up, stabilization, and definitive treatment (Figure 2).

## Conclusion

Diagnosis and treatment of malignancy induced LA represents a therapeutic challenge in the intensive care setting. We propose a stepwise approach to ensure thorough and fast diagnostic work-up, stabilization and establishment of definitive treatment. Further research is warranted to define evidence-based guidelines and improve outcomes in this vulnerable population.
